# Enhanced antitumor efficacy and reduced toxicity of Abnormal Savda Munziq on tumor bearing mice treated with chemotherapy

**DOI:** 10.18632/oncotarget.21563

**Published:** 2017-10-06

**Authors:** Tao Yang, Wenhui Shi, Zukereguli Wumaierniyazi, Lianlian Shan, Weiwei Miao, Guizhen Wu, Bin Zhou, Abdryim Yusup, Halmurat Upur, Ainiwaer Aikemu

**Affiliations:** ^1^ Department of Drug Analysis, Faculty of Pharmacy, Xinjiang Medical University, Urumqi 830011, China; ^2^ Department of Animal Experiment, Urumqi General Hospital of Lanzhou Military, Urumqi 830011, China; ^3^ Central Laboratory of Xinjiang Medical University, Urumqi 830011, China; ^4^ Traditional Uighur Medicine and Pharmacology, Xinjiang Medical University, Urumqi 830011, China

**Keywords:** Abnormal Savda Munziq, cervical cancer, 5-FU

## Abstract

Previous research has demonstrated the anti-tumor properties of Abnormal Savda Munziq (ASMq), a traditional Uyghur compound herbal medicine. The effects of ASMq on cervical carcinomas in U27 tumor-bearing mice is investigated, the effect of adding Fluorouracil (5-FU) is also assessed in this paper. The results demonstrate that ASMq and 5-FU significantly inhibited the proliferation of U27 cells in a time-dependent and dose-dependent manner. Evaluating the interactions between ASMq and 5-FU on U27 cell growth yields a combination index (CI) < 1 in different time periods, suggesting a synergistic effect between the two drugs *in vitro*. Nuclear magnetic resonance (NMR) analysis demonstrates that ASMq can inhibit enhanced lipid metabolism in tumor mice, enhance the glutamine content, promote lymphocyte and macrophage proliferation, and increase tumor necrosis factor(TNF-α) and interleukin(IL) production, which can enhance the effect of 5-FU on the inhibition of tumors. Also ASMq can reduce the content of ALT and AST in serum. Increased SOD, GSH-Px, and decreased the content of MDA in liver tissue. ASMq has a synergistic effect on liver and tumor pathology, as well as tumor inhibition rate. In addition, ASMq can also enhance the body's antioxidant capacity and improve the body's metabolism, and reduce 5-FU's toxic side effects.

## INTRODUCTION

Cervical cancer represents one of the most common types of malignant tumor known to gynecological specialists. It has the highest prevalence and causes the greatest mortality in the high-risk cervical cancer group of women in Xinjiang, and it represents the leading cause of death for women in China's minority populations [1, 2]. Chemical medication therapy remains the most common cancer treatment method in both Chinese and Western medicine, but chemotherapy drugs used to treat tumors inhibit immune function and cause visceral toxic side effects [3–6]. Therefore, improving the effectiveness and decreasing the toxicity of chemotherapy are necessary to the future of medicine.

Uyghur medicine is an important part of traditional medicine, and the Uyghur people struggle with both disease and the established, mainstream medical system. The source of traditional Uighur medicine (TUM) can be traced to ancient Graeco-Arab medicine and has been used to diagnose, treat and prevent illnesses for more than 3,000 years in Xinjiang, China [7, 8]. Hilit (humor) theory constitutes the primary theoretical system in Uyghur medicine. Kan, Phlegm, Safra, and Savda serve as the fundamental components of hilit theory. Uyghur medicine posits that Abnormal Savda causes tumors and other diseases. Uyghur medicine employs mature/clear therapy to correct humoral disorders and treat abnormal savda, and Abnormal Savda Munziq (ASMq) granules are among the most commonly used treatment compound medicine [9–12]. Previous research has shown that ASMq demonstrates an obvious inhibitory effect on liver cancer and breast cancer, as its scavenging hydroxyl free radicals prevent oxidative damage to DNA and induce apoptosis in lymphocyte tumor cells. However, there is much less existing research regarding different attenuated synergies of ASMq in tumor chemotherapy. Metabolomics represents an important component of systems biology, which utilizes qualitative and quantitative monitoring of an organism or cell in order to identify all metabolites of low molecular weight both inside and outside the cell. This study employs metabolomics to observe the effects of ASMq with fluorouracil (5-FU) and attenuated synergies on cervical carcinomas in U27 tumor-bearing mice. We hope that this study will provide an experimental basis for further clinical applications of ASMq.

## RESULTS

### Effects of ASMq and 5-FU on the proliferation of U27 cells

**Figure 1:**
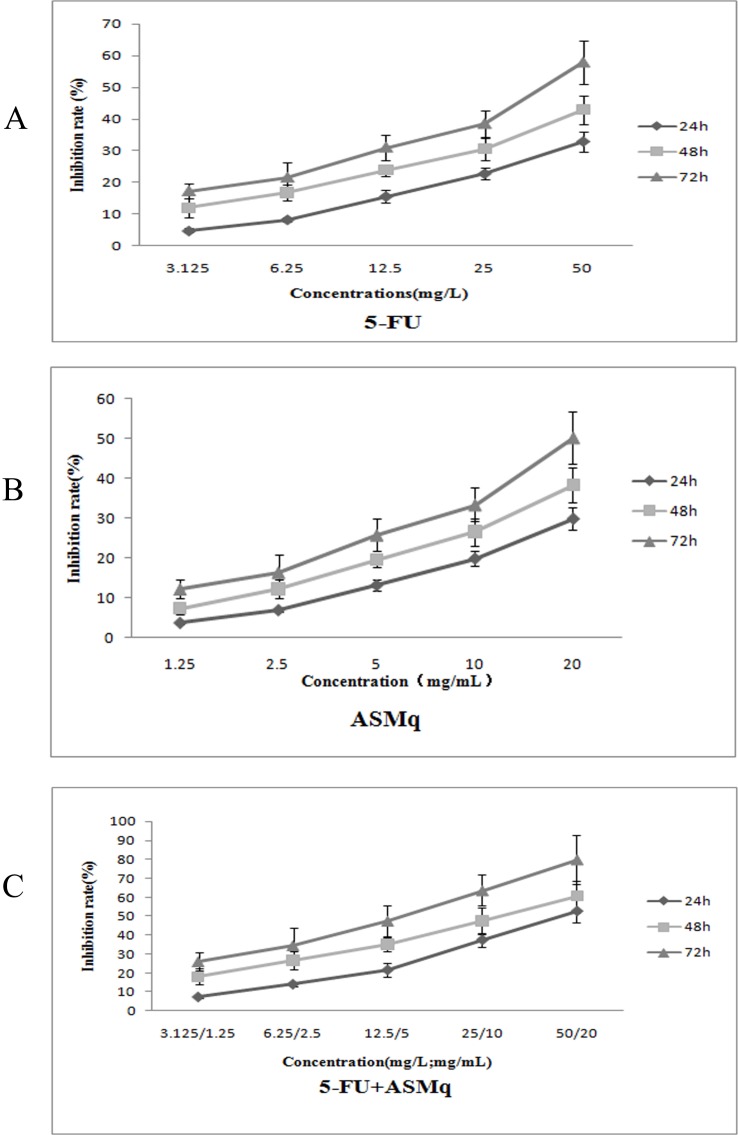
Inhibitory effect of ASMq or/and 5-fluorouracil on the growth of U27 cells **(A)** Growth inhibition rate of U27 cells following 24, 48 or 72 h treatment with increasing concentrations of 5-fluorouracil. **(B)** Growth inhibition rate of U27 cells following 24, 48 or 72 h treatment with increasing concentrations of Abnormal Savda Munziq (ASMq). **(C)** Growth inhibition rate of U27 cells following 24, 48 or 72 h treatment with increasing concentrations of 5-fluorouracil combined with Abnormal Savda Munziq (ASMq).

Compared with the control group, there are significant inhibitory effects on the proliferation of U27 cells with treated different concentration of ASMq and/or 5-FU, and the effects showed a time- and dose-dependent manner (Figure 1). According to calculation, the IC_50_ of 5-FU were 110.12 mg/mL (24 h), 81.42 mg/L (48 h), and 35.42 mg/L (72 h), and IC_50_ of ASMq were 52.45 mg/mL (24 h), 36.78 mg/mL (48 h), 21.12 mg/mL (72 h). However, when treated with two drugs simultaneously, the IC_50_ values for ASMq were fell to 18.13 mg/mL(24 h), 12.34 mg/mL(48 h), and 6.24 mg/mL(72 h), respectively. The IC_50_ values for 5-FU were fell to 46.03 mg/mL(24 h), 26.73 mg/mL(48 h), and 14.21 mg/mL(72 h), respectively. As shown in Table 1, according to the IC_50_ values of U27 cells treated with ASMq and 5-FU, combination index (CI) at different time points (24h, 48h, 72h) was less than 1. The results showed that ASMq could significantly increase the inhibitory effects on proliferation of U27 cells treated with 5-FU. Furthermore, owing to the CI was less than 1, it could be proved that there was a synergistic effect between these two drugs.

**Table 1:** Analysis of the interaction between the inhibition effect of ASMq in combination with 5-fluorouracil on cell growth by combination index

Times(h)	Combination index	*P* value
24	0.7041±0.1112	<0.05
48	0.6141±0.1041	<0.05
72	0.5243±0.0914	<0.05

### Growth inhibition of transplanted cervical cancer by ASMq combined with 5-FU

As shown in Table 2, the inhibition rate for tumors in the 5-FU+ASMq.H group, 5-FU+ASMq.M group, 5-FU+ASMq.L group, and 5-FU group were 79.17%, 89.63%, 68.43%, and 66.48% respectively, all of which indicate clear inhibitory effects. Compared with the model group, the tumors in the 5-FU group and the 5-FU with high/median/low dose of ASMq groups had significantly decreased weights (*P*<0.05). Compared with the 5-FU group, the 5-FU+ASMq.H and 5-FU+ASMq.M groups were also significantly decreased weights (*P*<0.05).

**Table 2:** Inhibitory effect of ASMq on the growth of transplanted cervical cancer tumors in mice when combined with 5-FU ($\bar{x}\pm s$)

Group	Dosage /(g/kg)	Start animals/ end animals	Tumor weight /(mg)	Inhibition rate /%
Control	-	10/10	0.000±0.000	-
Model	-	10/10	2682.5875±256.7856^b^	-
5-FU	0.03	10/10	899.0875±282.6327^a^	66.48
5-FU +ASMq.H	8	10/10	558.7875±342.0630^ab^	79.17
5-FU +ASMq.M	4	10/10	278.2875±153.0699^ab^	89.63
5-FU +ASMq.L	2	10/10	846.8750±372.0095^a^	68.43

VS. Model group, ^a^*P*<0.05;VS. 5-FU group, ^b^*P*<0.05.

**Figure 2:**
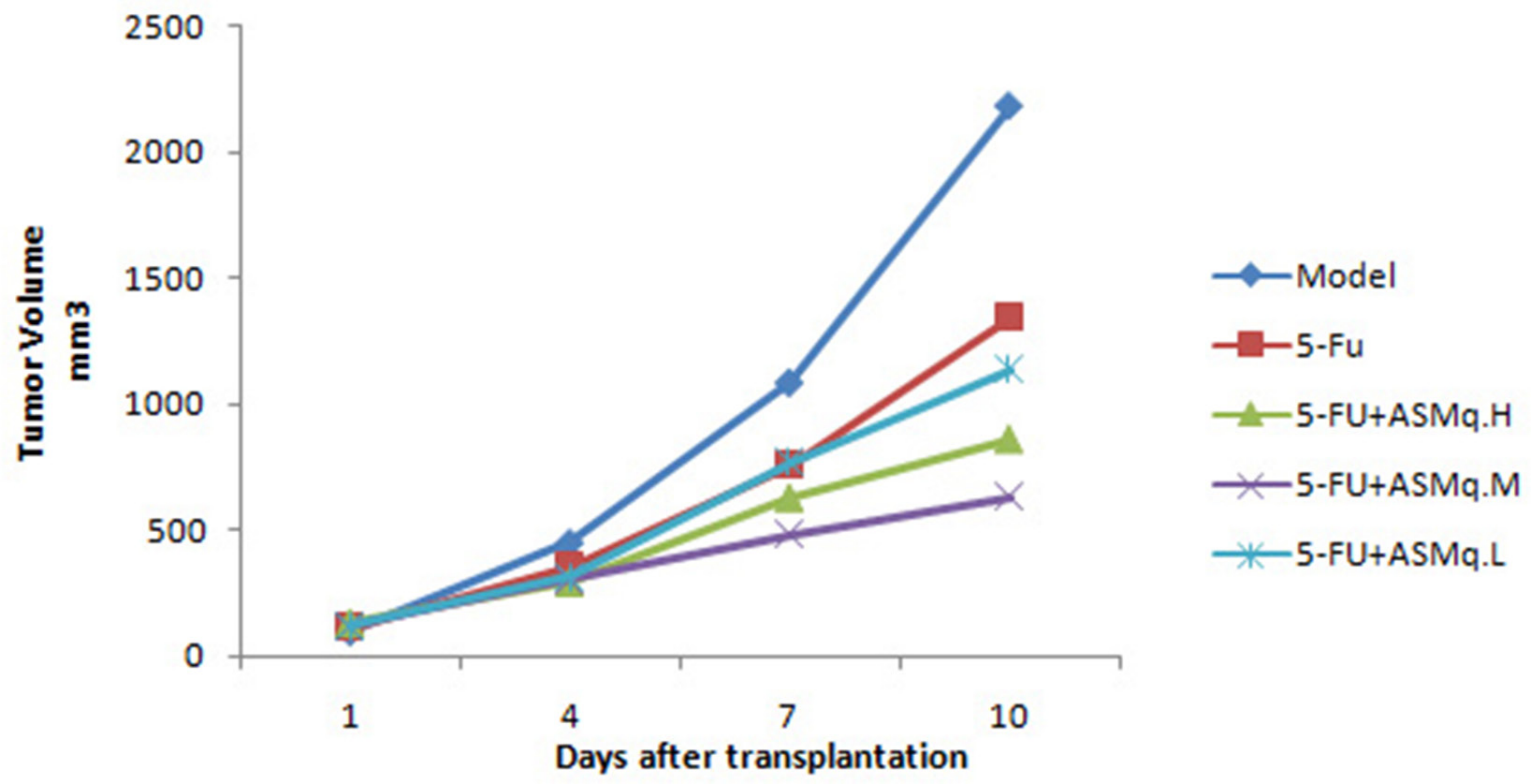
Tumor growth curve

As shown in Figure 2, after treatment with ASMq and 5-FU, the average tumor volume was less than in the 5-FU group, and the difference was statistically significant (*P*<0.05).

### ^1^H-NMR spectrum results of mouse serum samples

**Figure 3:**
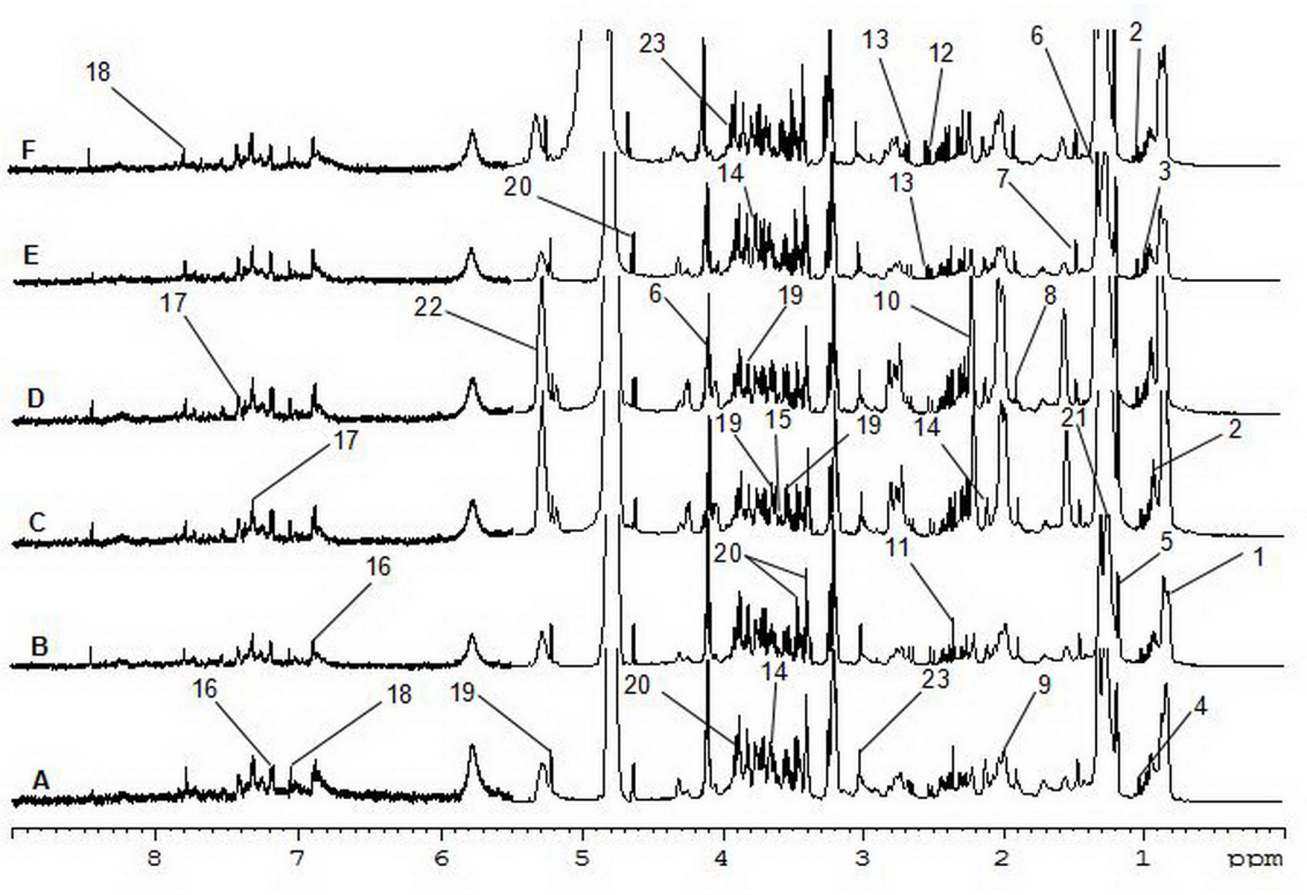
600 MHz ^1^H-NMR Spectra of serum **(A)** control group; **(B)** model group; **(C)** 5-FU group; **(D)** ASMq.L group; **(E)** ASMq.M group; **(F)** ASMq.H group.

**Figure 4:**
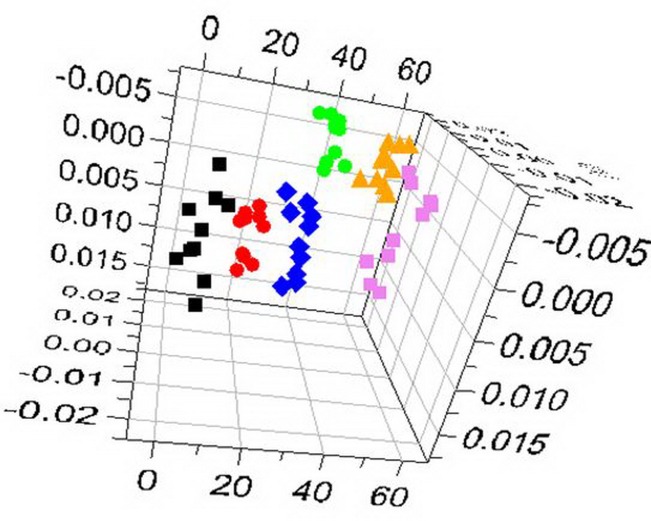
OPLS-DA 3D plots of ^1^H-NMR spectra 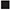
control group;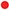 model group;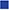 5-FU group;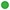 ASMq.L group;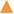 ASMq.M group; 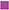
ASMq.H group.

Figure 3 shows ^1^H-NMR spectrum results for mouse serum samples from all groups. The ^1^H-NMR spectrum values after subsection integration were analyzed through PLS-DA and presented as three-dimensional plots (Figure 4). In this analysis, R^2^X=0.78, R^2^Y=0.22, and Q^2^=0.19. As shown in Figure 3, each group had a different distribution region, indicating that the contents of metabolites in the mouse serum were remarkably different among the different groups. The correlation coefficient between the two groups was obtained by OPLS-DA and used to determine the chemical shifts of differential metabolomics. The degree of variation was determined by the chemical shift in conjunction with the correlation coefficient.

The differential metabolites in the serum samples from different groups were determined by their ^1^H-NMR spectra, and the results are listed in Tables 3 and 4. A number of significantly different metabolites were identified when the different groups were compared with the control group. In the model group, for example, positive correlation coefficients indicate that the metabolites had higher levels than those in the control group, while negative correlation coefficients indicate decreased levels of metabolites compared with the control group. Additional comparisons between groups were performed according to the same analytical strategy.

**Table 3:** Otherness metabolites of mice serum in each group

Order number	Metabolite	Chemical shift(ppm)	Assignment
1	Lipids	0.85(m)	CH_3_(CH_2_)_n_
2	Isoleucine	0.93(t), 1.00(d)	δ-CH_3_, β-CH_3_
3	Leucine	0.97(d)	δ-CH_3_
4	Valine	1.03(d)	α-CH_2_
5	β-Hydroxy butyric acid	1.19(d)	γ- CH3
6	Lactate	1.32(d), 4.11(q)	CH3, CH
7	Alanine	1.46(d)	CH_3_, α-CH
8	Acetate	1.91(s)	CH_3_
9	Glycoprotein	2.03(s)	NHCO-CH_3_
10	Acetone	2.22(s)	CH_3_
11	Pyruvate	2.36(s)	CH_3_
12	Carnitine	2.44(dd)	CH_2_(COO)
13	Citrate	2.52(d), 2.66(d)	CH_2_, CH_2_
14	Glutamine	2.13(m), 3.76(m), 3.69(t)	halfβ-CH_2_, α-CH
15	Glycine	3.56(s)	CH_2_
16	Tyrosine	6.89(d), 7.18(d)	α-CH, H_3_/H_5_, H_2_/H_6_
17	Phenylalanine	7.32(m), 7.42(m)	H_2_/H_6_, H_4_, H_3_/H_5_
18	1-methylhistidine	7.05(s), 7.78(s)	H_4_, H_2_
19	a-glucose	3.53(dd), 3.72(d), 5.23(d), 3.83(m)	C-H_2_, halfCH_2_-CH_6_, C-H_1_, C-H_6_
20	β-glucose	3.23(dd), 3.40(t), 3.47(ddd), 4.64(d)	C-H_4_, C-H_5_, halfCH_2_-CH_6_, C-H_1_, C-H_3_
21	LDL	1.26(m)	CH_3_CH_2_(CH_2_)_n_
22	Unsaturated lipid	5.28(m)	CHCH_2_CH_2_
23	Creatine	3.03(s), 3.93(s)	CH_3_, CH_2_

s is singlet; d is doublet; t is triplet; q is quartet; m is multiplet; dd is doublet of doublets; ddd is doublet of doublets of doublets.

**Table 4:** Difference correlation coefficients of otherness metabolites in mice serum

Order number	Metabolite		Normal control group compared with the model group	model group compared with the 5-FU group	model group compared with the ASMq.L group	model group compared with the ASMq.M group	model group compared with the ASMq.H group	5-FU group compared with the ASMq.L group	5-FU group compared with the ASMq.M group	5-FU group compared with the ASMq.H group
		R^2^X	0.79	0.7	0.84	0.78	0.71	0.85	0.74	0.7
		Q^2^	0.34	0.3	0.86	0.83	0.51	0.79	0.67	0.6
1	Lipids		0.62	-0.74	-0.71	-0.65	-0.66	-0.81		
2	Isoleucine		-0.6			0.74			0.62	
3	Leucine					0.81			0.76	
4	Valine		-0.71			0.87			0.66	
5	β-Hydroxy butyric acid		0.74			0.66			0.67	
6	Lactate		-0.72							-0.81
7	Alanine		-0.63			0.63	0.67		0.61	0.72
8	Acetate		-0.73			0.74			0.89	
9	Glycoprotein		0.63					-0.81		
10	Acetone		0.6		-0.72	-0.92	-0.72	-0.76	-0.96	-0.82
11	Pyruvate		-0.62			0.73			0.94	0.73
12	Carnitine		0.72					-0.68		
13	Citrate		-0.73			0.77	0.58		0.65	
14	Glutamine		-0.75			0.67	0.62		0.67	
15	Glycine		-0.59					0.8	0.85	0.88
16	Tyrosine					0.72		-0.81	0.84	
17	Phenylalanine					0.8		0.58	0.71	
18	1-methylhistidine		-0.77		0.59	0.86	0.62		0.65	
19	a-glucose				0.6	0.69		0.73	0.63	
20	β-glucose			-0.64		0.71		0.77	0.75	0.67
21	LDL		0.65		-0.6	-0.91	-0.63	-0.95	-0.85	-0.87
22	Unsaturated lipid		0.63		-0.63	-0.72	-0.63	-0.69	-0.65	-0.6
23	Creatine		-0.74	-0.6		0.62	0.6	0.72	0.73	0.65

Lipids, glycoprotein, acetone, carnitine, low density lipoprotein (LDL), and unsaturated lipids were elevated in serum samples from the model group compared with the control group, while a variety of serum metabolites were remarkably reduced, including isoleucine, valine, lactate, alanine, acetic acid, pyruvate, citric acid, glutamine, glycine, 1-methylhistidine, and creatine. These differences were statistically significant (*P*<0.05). The serum level of lipids, glucose, and creatine were lower in the 5-FU group than in the model group (*P*<0.05). Moreover, compared with the model group, the levels of lipids, acetone, LDL, and unsaturated lipids were significantly decreased in the groups treated with all doses of ASMq in combination with 5-FU (*P*<0.05). The levels of 1-methylhistidine and glucose were increased in the ASMq.L group compared with the model group (*P*<0.05). Isoleucine, leucine, valine, β-Hydroxy butyric acid, alanine, acetic acid, pyruvate, citric acid, glutamine, tyrosine, phenylalanine, 1-methylhistidine, glucose, and creatine were higher in the ASMq.M group than in the model group (*P*<0.05). The levels of alanine, citric acid, glutamine, 1-methylhistidine, and creatine were higher in the ASMq.H group than in the model group (*P*<0.05). Meanwhile, different doses of ASMq in combination with 5-FU resulted in a remarkable increase of glycine, glucose, and creatine in serum compared with the 5-FU group, as well as significant reductions in the contents of acetone, LDL, and unsaturated lipids (*P*<0.05). Compared with the 5-FU group, the levels of lipids, glycoprotein, pyruvate, carnitine, and tyrosine decreased while phenylalanine and glucose increased in the ASMq.L group in combination with 5-FU (*P*<0.05). The levels of isoleucine, leucine, valine, β-Hydroxy butyric acid, alanine, acetic acid, pyruvate, citric acid, glutamine, tyrosine, phenylalanine, 1-methylhistidine, and glucose increased significantly in the ASMq.M group in combination with 5-FU, as compared with the 5-FU group (*P*<0.05). The level of lactate in the ASMq.H group in combination with 5-FU decreased notably compared with the 5-FU group, but alanine and pyruvate increased (*P*<0.05).

### Effect of 5-FU combined with ASMq on serum IL-2, TNF-α content of U27 bearing mice

Compared with the control group, serum IL-2, TNF-α contents were decreased for mice in model group. Serum IL-2, TNF-α content were lower in 5-FU group. On the contrary, serum IL-2 and TNF-α levels were increased for mice in the 5-FU combined with high/median dose of ASMq groups. In 5-FU group showed lower level of serum IL-2 and TNF-α than those in model group. At the same time, the 5-FU group showed increased level of serum IL-2 and TNF-α contents. In 5-FU with high/median/low dose of ASMq groups, levels of serum IL-2 and TNF-α contents were increased. Compared with the 5-FU groups, in 5-FU combined with high/median/low dose of ASMq groups also demonstrated increased level of serum IL-2 and TNF-α. Detailed data are illusrated in Table 5.

**Table 5:** Effect of 5-FU combined with ASMq on serum IL-2, TNF-α content of U27 bearing mice ($\bar{x}\pm s$, n=10)

Group	Dosage (g/kg)		IL-2 (U/mg prot)	TNF-α (pg/ml)
	ASMq	5-FU		
Control	-	-	16.9813±0.8103	18.0654±1.2321
Model	-	-	14.9947±0.7124^ac^	15.6427±0.9671^ac^
5-FU	-	0.03	13.7231±0.7123^ab^	14.8337±1.2120^ab^
5-FU+ASMq.H	8	0.03	19.2434±1.0271^abc^	21.9538±0.9837^abc^
5-FU+ASMq.M	4	0.03	22.9654±1.2587^abc^	26.9731±0.7454^abc^
5-FU+ASMq.L	2	0.03	17.8847±0.8124^bc^	18.7351±1.3423^bc^

VS control group, ^a^*P*<0.05; VS model group, ^b^*P*<0.05, VS 5-FU group, ^c^*P*<0.05.

### Effect of 5-FU combined with ASMq on serum IL-2, TNF-α content of U27 bearing mice

Compared with the control group, serum IL-2, TNF-α contents were decreased for mice in model group. Serum IL-2, TNF-α content were lower in 5-FU group. On the contrary, serum IL-2 and TNF-α levels were increased for mice in the 5-FU combined with high/median dose of ASMq groups. In 5-FU group showed lower level of serum IL-2 and TNF-α than those in model group. At the same time, the 5-FU group showed increased level of serum IL-2 and TNF-α contents. In 5-FU with high/median/low dose of ASMq groups, levels of serum IL-2 and TNF-α contents were increased. Compared with the 5-FU groups, in 5-FU combined with high/median/low dose of ASMq groups also demonstrated increased level of serum IL-2 and TNF-α. Detailed data are illusrated in Table 5.

### The effects of 5-FU combined with Abnormal Savda Munziq on the expression of ALT, AST in mice serum

Compared with the control group, the expression of ALT and AST in mice in the 5-FU group and groups of 5-FU+ASMq at different doses, increased significantly (*P*<0.05). Compared with the model group, the content of ALT and AST in mice in the 5-FU group and groups of 5-FU+ASMq at different doses, increased significantly (*P*<0.05). Compared with the 5-FU group, the expression of ALT and AST in mice in groups of 5-FU+ASMq decreased significantly (*P*<0.05). These results are shown in Table 6.

**Table 6:** The effects of 5-FU combined with Abnormal Savda Munziq on the expression of ALT, AST in mice serum ($\bar{x}\pm s$)

Group	Dosage (g/kg)		ALT (pg/ml)	AST (pg/ml)
	ASMq	5-FU		
Control	-	-	83.758±1.103	47.832±0.601
Model	-	-	79.875±3.114^c^	47.980±0.521^c^
5-FU	-	0.03	506.864±22.061^ab^	237.145±6.424^ab^
5-FU+ASMq.H	8	0.03	198.132±14.451^abc^	147.173±7.455^abc^
5-FU+ASMq.M	4	0.03	121.165±14.176^abc^	80.724±1.453^abc^
5-FU+ASMq.L	2	0.03	397.794±35.407^abc^	217.425±8.147^abc^

VS control group, ^a^*P*<0.05;VS model group, ^b^*P*<0.05; VS 5-FU group, ^c^*P*<0.05.

### The effect of 5-FU combined with ASMq on the liver of mice bearing U27 tumor

As shown in Table 7, Compared with the control group, the expression of SOD and GSH-Px in liver tissues in the 5-FU group, decreased significantly (P<0.05), and increased MDA. Compared with the 5-FU group, the expression of SOD and GSH-Px in liver tissues in groups of 5-FU+ASMq increased significantly (P<0.05), and decreased MDA

**Table 7:** Effect of 5-FU combined with ASMq on the liver of mice bearing U27 tumor ($\bar{x}\pm s$)

Group	Dosage (g/kg)		SOD (U/mg prot)	GSH-Px (U/mg prot)	MDA (nmol/mg prot)
	ASMq	5-FU			
Control	-	-	90.678±1.786	134.432±10.145	5.227±0.124
Model	-	-	73.378±2.446 ^ac^	101.045±4.123^ac^	9.996±0.785^ac^
5-FU	-	0.03	57.456±3.651 ^ab^	91.147±2.229^ab^	13.457±0.716^ab^
5-FU+ASMq.H	8	0.03	101.345±3.245 ^abc^	116.021±2.476^abc^	6.978±0.564^abc^
5-FU+ASMq.M	4	0.03	120.154±2.667 ^abc^	149.554±3.174^abc^	5.013±0.382^bc^
5-FU+ASMq.L	2	0.03	100.045±4.4786^abc^	106.124±2.313^abc^	7.453±0.768^abc^

Note: VS control group, ^a^*P*<0.05;VS model group, ^b^*P*<0.05; VS 5-FU group, ^c^*P*<0.05.

### Pathological examination of liver cells from all experimental groups

**Figure 5:**
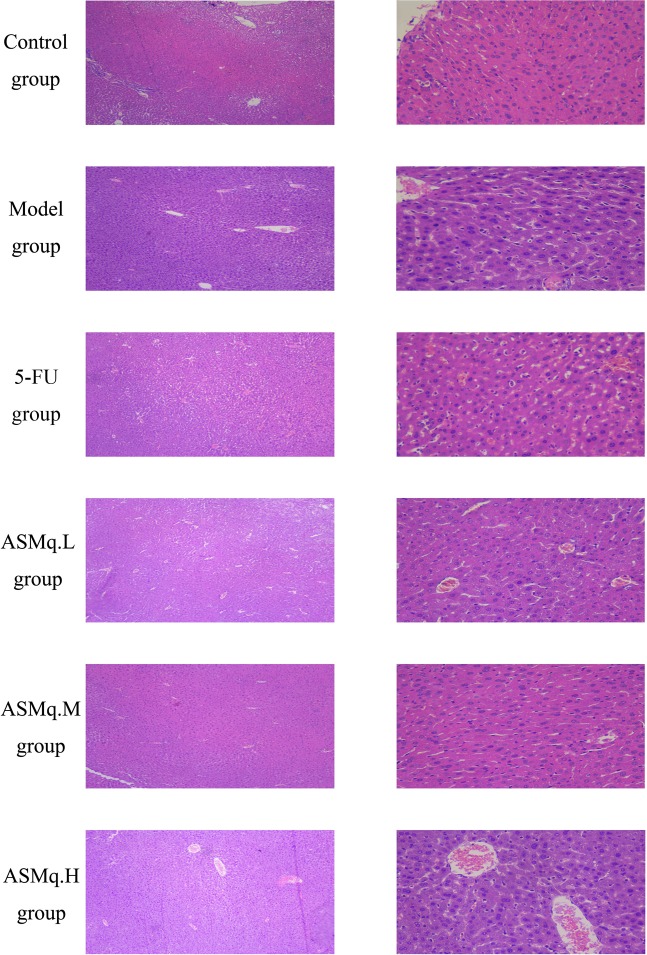
Liver pathology examination

Pathological changes in the livers of all experimental groups were analyzed by HE staining. The histological sections shown in Figure 5 make it clear that the livers in the control group possessed a normal structure, without any lesions. The typical hepatic lobule structure was still present, the morphology of the hepatocytes was normal, the hepatic cords were radially arranged, and a minimal amount of blood was observed in the vein. The livers from the model group showed a normal structure without any lesion, and the hepatic cords were radially arranged. For mice in the 5-FU group, the spindle structure of the hepatocytes was still present, but swollen hepatocytes and a small number of infiltrated inflammatory cells were observed near the veins; furthermore, dilation of the central veins was reduced compared with the model group, but serious congestion was still present. In the livers from the ASMq.M group, the spindle structure of the hepatocytes was lost, no dilation of the central veins was observed, and slight congestion in the central veins and hepatic sinusoids was detected. Occasional swollen hepatocytes were also noted in this group. In the livers from the ASMq.H group, the spindle structure of the hepatocytes was barely detectable, and prominent dilation and moderate congestion were observed in the central veins. Slight congestion appeared in the hepatic sinusoids and swollen hepatocytes.

### Pathological examination

**Figure 6:**
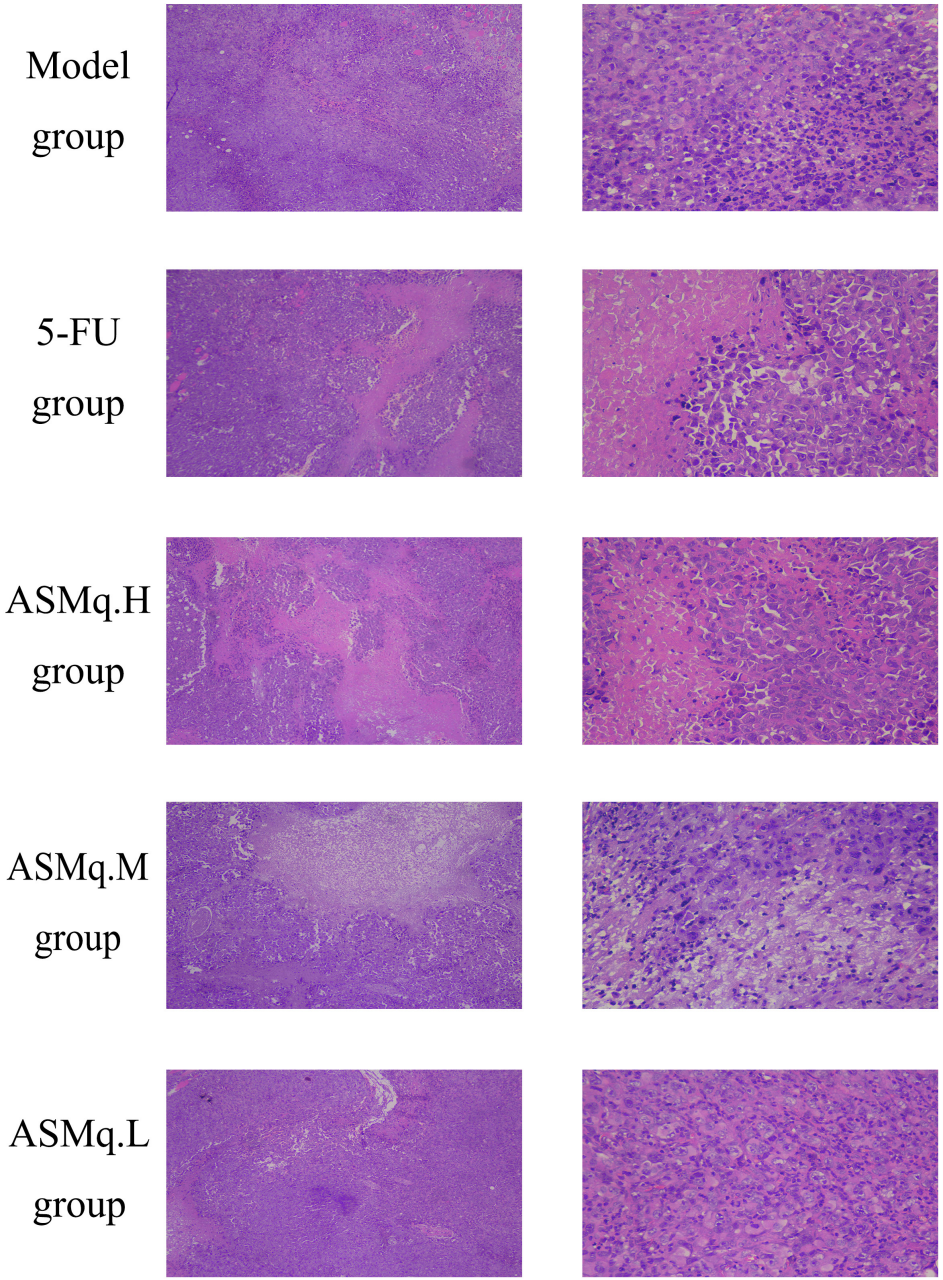
Tumor pathology examination

As shown in Figure 6, The model group showed diffused distribution of tumor cells, where the cells showed different sizes with some cells had mild edema. Also model group showed cells shows diffused distribution, cell sizes ranging in a wide spectrum, shades, even with mild edema in some cells, as well as interstitial infiltrated inflammatory cells.

The 5-FU group showed the tumor cells were necrosis, and partly showed mild edema. Also 5-FU group showed the contour of the tumor cells were still clear, and the size of the tumor cells were visible.

The 5-FU combined with high dose of ASMq group examined visible tumor tissue necrosis. The vast majority of cells showed clear outline, while some tumor cells dissolve fine granular, a small amount of tumor cells showed mild edema. The 5-FU combined with high dose of ASMq group displayed under high magnification tumor cells can be seen in different sizes, with different shapes, irregular arrangement, partial nuclear condensation, interstitial hemorrhage and edema.

The 5-FU combined with middle dose of ASMq groups Under low magnification showed the tumor tissue was necrosis or necrotic, and the cells disintegrated at the edge of necrosis was granular and interstitial edema. Under high magnification, showed patchy necrosis of the tumor cells’ outlines were clear, cell sizes were different, nuclear disappearance, interstitial edema, occasionally scattered in the inflammatory cell infiltration.

Treated with 5-FU combined with ASMq low dose group, a large number of dense tumor cells were found in the necrosis of the tissue, which was mainly composed of liquefied necrosis. Uner high magnification, it showed that some tumor cells were round, same size, a large number of tumor cells disintegrated granular, magnified interstitial edema.

## DISCUSSION

Currently, chemotherapy is a primary means of treating cancer. However, during their course of treatment, chemotherapy drugs can inhibit immune function and induce organ toxicity, among other side effects; drug resistance also frequently develops. Therefore, the application of synergistic attenuation drugs in chemotherapy regimens has important clinical significance for improving the survival rate and quality of life for cancer patients. Abnormal Savda Munziq is a Uyghur compound medicine comprised of ten separate drugs including *Adiantum capillus-veneris L*, *Alhagi pseudalhagi (Bieb.) Desv*, *Anchusa italica Retz*., *Cordia dichotoma G.Forst*, *Euphorbia humifusa Willd*, *Foeniculum vulgare Mill*, *Glycyrrhiza glabra L*, *Lavandula angustifolia Mill*, *Melissa officinalis L*, and *Ziziphus jujuba Mill*. This mixture has been widely used in the treatment of a variety of tumors in Uyghur medicine. Studies have shown that certain drugs that make up Abnormal Savda Munziq prescription have significant anti-cancer behaviors. For example, *Glycyrrhiza glabra L* inhibit the growth of human hepatoma cell lines (Hep3β) [13] and breast cancer cell lines (MCF-7) [14], and has been shown to induce apoptosis in human gastric cancer cell lines (MGC-803) [15]. *Ziziphus jujuba* can reduce the survival rate of HepG2 *in vitro*, induce apoptosis, and block the cell cycle [16]. In addition, a low concentration (50μg/mL) of total phenol of Abnormal Savda Munziq exhibited no action against squamous cell carcinoma SiHa, but is thought to increase the inhibition rate of cell proliferation when used in combination with the chemotherapy drugs cisplatin or docetaxel [17].

The method of nuclear magnetic resonance (NMR) has been applied to cancer research, which has been recognized by the most people [18, 19]. It is well known that the small molecule metabolites are associated with tumor *in vivo* [20], and we have studied the mechanism of anti tumor *in vivo* by using the method of ^1^H-NMR [8, 21]. On the basis of previous studies, we used the method of ^1^H-NMR in the study on the synergism and attenuation action of ASMq combined fluorouracil.

As early as 1956, Warburg discovered that malignant tumor cells often behave differently from normal cells; specifically, under aerobic conditions, tumor cells are mainly powered through the glycolysis pathway [22]. Because the energy efficiency of the glycolysis pathway is very low, an increase in fat metabolism may be required in order for tumor cell proliferation to maintain a high-speed power supply. In this study, β-hydroxybutyric acid and acetone are intermediates produced by the β-oxidation of fatty acids in liver mitochondria. Carnitine is an essential transporter for the activated acyl CoA from the cytoplasm to mitochondria during β-oxidation of fatty acids. With the help of a series of enzymes, acetone can be converted into pyruvate or lactic acid, or can be excreted during urination or exhalation. However, excessive dosages of β-hydroxybutyric acid can lead to ketoacidosis [23–25]. For mice in the tumor model group, the β-hydroxybutyric acid and carnitine levels were elevated and the acetone level was reduced, indicating aggravated β-oxidation of fatty acids in tumor mice. Creatine has been shown to lower cholesterol and triglycerides in the body. Lipids, unsaturated lipids, and LDLs are all products of fat decomposition. For mice in the tumor model group, the level of lipids, unsaturated lipids, and LDLs all increased, indicating enhanced fat mobilization, which may have been related to adecreased level of creatine. After a combination of Abnormal Savda Munziq with Fluorouracil (5-FU) was administered, the serum levels of β-hydroxybutyric acid and carnitine decreased whereas that of acetone increased. The levels of lipids, LDLs, acetone, and unsaturated lipids also decreased. These results demonstrated that abnormal savda munziqinhibited enhanced fat mobilization, reduced tumor cell supply, and thereby inhibited tumor cell proliferation and growth.

Immune function is closely related to the occurrence and development of tumors. Usually, malignant tumor patients exhibit low immune function, which is more significant during chemotherapy. Glutamine plays an important role in lymphocyte secretion, proliferation, and function maintenance. When lymphocytes proliferate and differentiate heavily after being stimulated by antigens, glutamine is an important precursor of nucleotide synthesis and is also an important energy source for lymphocytes. Glutamine can promote mitosis and differentiation of lymphocytes and macrophages, which increases the production of cytokines, such as tumor necrosis factor (TNF) and interleukin (IL), and the synthesis of phospholipid mRNA. Branched-chain amino acids (BCAAs), including valine, leucine, and isoleucine, are important nutritional supplements that can stimulate the proliferation of monocytes, regulate the secretion of cytokines, and promote the development of Th1-type immune response. An imbalance in the proportion of BCAAs can lead to immunological damage. Furthermore, adeficiency of valine can lead to abnormal differentiation and maturation of dendritic cells. For mice in the tumor model group and 5-FU group, the levels of glutamine, TNF-α, IL-2, and BCAAs were all lower than those of the control group, indicating that both the tumor mice and chemotherapy mice were immunocompromised. Conversely, the combination of Abnormal Savda Munziq with 5-FU increased the levels of glutamine, TNF, IL, and BCAA in serum, demonstrating that the Abnormal Savda Munziq inhibited tumor growth by regulating immune function.

Liver is the main site of drug metabolism in the human body, however, in the treatment of cancer, chemotherapy drugs on the liver are able to produce different levels of influence, and what's more, they also can lead to death. Most of 5-FU were metabolized in the liver, and only about 15% of 5-FU were eliminated from the human body as the original urine. Moreover, Long term application of 5-FU can lead to liver injury [26]. Alanine transaminase (ALT) and aspartate transaminase (AST) are mainly distributed in liver cells, and their increased levels are consistent with the degree of liver cell damage. Thus, they are the most commonly-used indicators of liver function. ALT is generally present in the soluble portion of liver plasma. Liver cell lesions may cause cell swelling, necrosis, or increased permeability of liver cell membranes, all of which can release ALT into the blood and increase the serum levels of ALT. AST is mainly present in liver mitochondria. Necrosis or severe liver damage can cause a significant increase in AST. Creatine is an important compound synthesized in the liver for energy storage and utilization. If it is synthesized using glycine as the foundation, it is phosphocreatine, which is a high-energy phosphate reserving ATP. Decreased phosphocreatine levels have indicated disordered energy metabolism in tumor model mice, and glycine is a metabolite negatively correlated with tumor growth [23]. In this study, serum creatine levels in the 5-FU group decreased significantly; the possible mechanism may have been related to side effects of 5-FU, whose toxicity may cause organ damageand subsequently, decreased synthesis of creatine in the liver.

A large number of harmful free radicals produced by the cancer patients’ body who taken chemotherapy drugs in, which resulted in a variety of side effects [27]. In the previous study we have found that the scavenging free radicals activity of the ASMq *in vitro* [28, 29]. Superoxide dismutase (SOD) and glutathione peroxidase (GSH-Px)are important antioxidant enzymes *in vivo* whose activities indicate the body's antioxidant and free radical scavenging capacity. Malondialdehyde (MDA) is an important product of lipid peroxidation *in vivo*. Its production is related to lipid peroxidation damage, and is also associated with enzyme activity of the antioxidant system. Therefore, the amount of MDA can indicate the degree of lipid peroxidation, indirectly indicating the degree of cell damage. 1-methylhistidine can be converted via desmethyl into histidine, which has an antioxidant effect. By maintaining its reserves, glutamine (GSH) can also enhance the antioxidative capacity of the body, protecting cells, tissues, and organs from free radical damage [30]. In this experiment, serum AST and ALT levels were significantly increased in the 5-FU group, serum glutamine and 1-methylhistidine levels were decreased, and SOD, GSH-Px, and MDA levels were increased in the liver tissue. These results exhibited that 5-FU is toxic to mice livers and can cause oxidative damage. Nevertheless, for the mice group using a combination of Abnormal Savda Munziq and 5-FU, serum AST and ALT levels were significantly decreased, serum glutamine and 1-methylhistidine levels were increased, while SOD, GSH-Px levels were increased and MDA level decreased in the liver tissues. These results demonstrated that Abnormal Savda Munziq can enhance the activity of antioxidant enzymes and inhibit the formation of lipid peroxides in mice undergoing chemotherapy, thereby eliminating free radicals and reducing liver oxidative damage. For verification, liver pathology confirmed the results.

We did a study on ASMq combined 5-FU *in vitro* by MTT method, and suggested that there was a synergistic effect between these two drugs. In the early stage of the study, we studied the anti-tumor effect of ASMq on tumor cells U27 *in vivo*, which showed that ASMq had a certain anti-tumor effect on cervical cancer cell line U27. The experimental study on synergistic toxicity *in vivo*, which compared with the group of fluorouracil, ASMq combined 5-FU group had shown as a good increasing inhibition rate, and verified by the study on histopathology to tumor tissue.

In summary, ASMq can regulate the body's metabolism through the immunoregulation function, scavenge free radical, reduce liver damage fluorouracil induced, antagonize the immune inhibition 5-FU induced, and its anti-tumor effect. Hence, these function suggested that ASMq is a compound drugs with multi target and pathway, and has synergism and attenuation action to 5-FU.

## MATERIALS AND METHODS

### Materials and instruments

This study used an AL204 analytical balance and PL602S electronic balance manufactured by the Mettler-Toledo Instrument Co., Ltd. (Shanghai, China). The hypothermal ultracentrifuge was from Beckman (USA), and the Inova600 NMR spectrometer was from Varian (USA). Topspin 2.0 software was from Bruker (Germany), and SIMCA-P+11 software was from Umetrics (Sweden). The -80°C ultra cold storage freezer was from Haier (China). The LEICA RM 2016 microtome was from Shanghai Wuxiang Instrument Co., Ltd. The HHB11420 electro-heating standing-temperature cultivator was from Shanghai Yuejin Medical Instrument Co., Ltd. The BHR-REL-T2 fluorescence microscope was from Olympus (Japan).

ASMq from lot no. 106060 was purchased from Xinjiang Ciconhabo Uyghur Medicine Co., Ltd., and 5-FU from lot no. 0912302 was purchased from Tianjing Jinyao Amino Acid Co., Ltd. Saline from lot no. 1311161 was purchased from Xinjiang Pharmaceutics of Sino Pharm, and D_2_O was purchased from Sigma-Aldrich (USA). Sodium chloride (NaCl), dipotassium hydrogen phosphate (K_2_HPO_4_), sodium dihydrogen phosphate (NaH_2_PO_4_), and 5 mm NMR tubes were all purchased from the Tianjin Guangfu Fine Chemistry Institute. Enzyme-Linked Immunosorbent Assay (ELISA) kit for Tumor Necrosis Factor Alpha (TNF-α) (Boster Biological Technology, Lot No. 241101041125) Enzyme-linked immunosorbent assay (ELISA) kit for Interleukin 2 (Boster biological technology, a batch 120101081121)

Enzyme-Linked Immunosorbent Assay (ELISA) kit for Aspartate transaminase (AST) (Nanjing Jiancheng Biological Technology Co. Ltd., Lot No. 201304010). Enzyme-Linked Immunosorbent Assay (ELISA) kit for Alanine aminotransferase (ALT) (Nanjing Jiancheng Biological Technology Co. Ltd., Lot No. 201304010). super-oxide dismutase (SOD) kit (Nanjing Jian cheng Bio-engineering Institute, batch number; 20130410); malondialdehyde (MDA) kit (Nanjing Jian cheng Bioengineering Institute, batch number: 20130409); glutathione per-oxidase (GSH-PX) kit (Nanjing Jian cheng Bio-engineering Institute, batch number: 201304010)

### Experimental animals and cell line

The present study protocol was approved by the Ethics Committee of Xinjiang Medical University (Ürümqi, China). All methods are in strict accordance with the Ethics Committee's approval document.

Sixty healthy Kunming mice (30 males and 30 females) of SPF grade were provided by the Animal Laboratory Center of Xinjiang Medical University (License No. SCXK(Xin)2011-0004). The specimens’ average body weight was 20±2 g. A cervical carcinoma U27 cell line was purchased from the Cell Bank of Wuhan University.

### MTT assay for the inhibition of U27 cell growth

The U27 cells were collected during logarithmic growth phase and made from a single cell suspension after digestion with trypsin (2.5g/L), and cell density was adjusted as 1.0 × 104 cells/well. Then 200μL cell suspension with 100μL medium were added in 96-well plates and cultured with 5% CO2 at 37°C. The experimental group was cultured with ASMq, 5-FU or ASMq+5-FU, respectively. The final concentrations of AMSq were 1.25, 2.5, 5, 10, and 20 mg/mL, the final concentrations of 5-FU were 3.125, 6.25, 12.5, 25 and 50 mg/L, and the final concentrations of ASMq+5-FU were 1.25+3.125, 2.5+6.25, 5+12.5, 10+25, and 20+25 mg/mL. Each concentration was repeated five wells, and the control group was added same volume medium without sample. After cultured 24, 48 and 72h, 20μL MTT (5 mg/mL) agent were added to each well and re-cultured 4h. Then 150μL DMSO agent were added after removing the liquid in each well. The absorbance at 570nm was measured. Inhibition rate of cell growth was measured according to the formula: inhibition rate (%) = [1- OD570 (experiment group)/OD570 (control group)] × 100. All experiments were made 3 times to get the average value. Samples (5-FU, ASMq and the combination) of the half maximal cell inhibitory concentration (IC50) was calculated and analyzed.

### Establishment of the tumor model and treatment with cervical carcinoma U27 cells

Sixty healthy Kunming mice were randomly divided into the following six groups: control group, model group, 5-FU group, 5-FU combined with a low dose of ASMq (5-FU+ASMq.L), 5-FU combined with a medium dose of ASMq (5-FU+ASMq.M), and 5-FU combined with a high dose of ASMq (5-FU+ASMq.H). Ascites (milky white) were collected under sterile conditions from a Kunming mouse treated with U27 cells for seven days, and the cells were then diluted with sterile saline to achieve a final concentration of 1.0×10^7^ cfu/mL. Cervical carcinoma U27 xenografts were established in all mice except for those in the control group by subcutaneously inoculating 0.2 mL of U27 of the ascite suspension into the left armpit of each mouse in the five experimental groups. The whole process was performed within 30 minutes in a sterile environment. Drug intervention was made 24 hours after the inoculation according to the following protocols: (1) Control group mice received an intraperitoneal injection of 0.4 mL saline, and they subsequently received saline at a dose of 0.2 mL/10 g per day by intragastric administration; (2) Model group mice received an intraperitoneal injection of 0.4 mL saline, and they subsequently received saline at a dose of 0.2 mL/10 g every other day by intragastric administration; (3) 5-FU group mice received an intraperitoneal injection of 5-FU at a dose of 25 mg/kg on the first day and every other day thereafter for a total of five administrations, and they subsequently received the same amount of saline every day by intragastric administration. The mice in the remaining three groups, (4) 5-FU+ASMq.L, (5) 5-FU+ASMq.M, and (6) 5-FU+ASMq.H, received an intraperitoneal injection of 5-FU at a dose of 25 mg/kg on the first day and every other day thereafter for a total of five administrations, and they subsequently received ASMq every day at a dose of 2 g/kg, 4 g/kg and 8 g/kg, respectively, by intragastric administration. All drug interventions lasted for 10 days. The mice were allowed ad libitum access to food and drinking water, and their body weights were measured every day.

### Growth inhibition of transplanted cervical cancer by ASMq combined with 5-FU

After after drug treatment, the length (A) and the shortest diameter (B) were measured in the tumor once every three days, and the tumor volume was calculated according to the formula V=AB^2^/2. The tumor growth curve was drawn for each group of tumor-bearing nude mice. After the last administration, the mice were sacrificed, and the tumors were immediately stripped and weighed. The tumor inhibition rate (IR) = (model group average tumor weight- experimental group average tumor weight) / average model group weight ^*^ 100%.

### Blood sampling, processing, and storage

Serum samples were collected from the mice in all groups on the day following the last drug administration. Blood was collected from the orbital cavity of the mice, stored at room temperature for 30 min, and centrifuged at 3000 rpm for 15 min at 4°C. The supernatant was collected and stored at -80°C.

### Pretreatment of serum metabolomic samples

The supernatants stored at -80°C were thawed at room temperature before analysis by NMR-hydrogen spectrum. Two hundred (200) μL serum was mixed with 400 μL buffer (0.3319 g K_2_HPO_4_, 0.067 g NaH_2_PO_4_, and 0.39 g NaCl, dissolved in 8 mL D_2_O and 32 mL H_2_O and mixed well to obtain 40 mL buffer), placed at room temperature for 10 min, and subsequently centrifuged at 10,000 rpm for 10 min. A total of 550 μL supernatant was transferred into a 5 mm NMR tube, labeled, and kept at 4°C for subsequent experiments.

### Determination of ^1^H-NMR in mouse serum and data interpretation

Hydrogen spectra were determined by INOVA 600 NMR spectrometer using the Carr-Purcell-Meiboom-Gill (CPMG) pulse sequence (RD-90°(τ- 180°-τ)n - ACQ). The test temperature was set at 25°C, and the NMR frequency was 599.95 MHz, with 128 scans collected and 32,768 data sampling points. The spectral width was 10,000 Hz, the average scanning time was 1.64 s per scan, and the delay was 2 seconds. The H_2_O peak was suppressed through a pre-saturation method. All ^1^H-NMR spectra were obtained from the same spectrometer for the purposes of interpreting and identifying the metabolomic results.

After NMR experiments, the data were imported into the Topspin 2.0 software for baseline and threshold calibration, which was set to a 5.233 ppm shift of the proton on glucose. Data between 0.5 ppm and 8.5 ppm were automatically integrated from 2,668 segments at 0.003 ppm intervals, and the region between 5.20 ppm and 4.66 ppm was removed in order to avoid confusion with H_2_O consumption in the concentrations of metabolites. The integrated values were then standardized. SIMCA-P+11 software was used for orthogonal partial least squares discriminant analysis (OPLS-DA), with R^2^X and Q^2^ used as the quality evaluation index of the model. R^2^X indicates the optimization degree of the model, R^2^Y represents the percentage of change in the variable Y, and the cross validation parameter Q^2^ describes the model's degree of accumulated prediction and indicates the authenticity of the predicted results. In this study, the metabolites-related correlation coefficient was used to verify the differential metabolites between the control group and the model group, with a test parameter of a=0.05. Pearson's product moment correlation coefficient |r|>0.576 (n=10) was used to test whether changes in metabolite content were significant. Metabolites with |r|>0.576 were considered statistically significant (P<0.05). Greater |r| values represent greater differences, and vice versa.

### Expression levels of TNF-α, IL-2, AST, ALT in mouse serum

After continuous treatment for 10 days, mice orbital blood was collected and kept at room temperature for 30 min, then centrifuged at 3000 rpm for 20 min before the supernatants were collected. The expression levels of TNF-α, IL-2 in mouse serum were determined according to the protocols of the corresponding kit.

### Measurements of SOD, GSH-Px and MDA in liver of U27 tumor bearing mice

Continuous administration lasted for 10 days after modeling. On the 10th day, the mice were sacrificed and had the liver and kidney removed and put it on the ice tray, all rinsed clean with saline solution of 4°C. Then they were weighed after being dried with filter paper. After that they were made into 10% (w/V) solution of tissue homogenate with 4°C saline solution in ice bath. Then this solution went through 3500 rpm centrifugation for 10 min at 4°C. Next the supernatant was taken to determine liver tissue homogenate and renal tissue homogenate protein content with Coomassie brilliant blue method, as well as level of SOD in liver homogenate and kidney homogenates, GSH PX and the content of MDA with colorimetric method (all operations strictly followed instructions provided from kit used).

### Pathological examination of mouse liver tissues

The mice were killed by cervical dislocation. After each mouse were killed, its liver was removed, washed with saline, and fixed with 10% formalin. The mouse livers were prepared in paraffin blocks. Each liver fixed with 10% formaldehyde was placed in 70% ethanol for 3 hours, 80% ethanol for 2 hours, 90% ethanol for 1.5 hours, and 100% ethanol for 1 hour, and then cleared in xylene for 30 min. The liver was submerged in melted paraffin at 54°C for 3 hrs, and the tissue-embedded paraffin was then solidified at room temperature.

To prepare the liver sections, tissue-embedded paraffin blocks were cut into 4 μm sections, which were extended on a surface of 30-40% ethanol and water at 38°C, placed onto glass slides, and incubated at 37°C overnight. The sections were then kept at room temperature.

The sections were de-waxed by incubation in xylene for 15 min for a total of three times, followed by serial rinses in 100% ethanol twice for 2 min each, in 95% ethanol for 2 min, in 80% ethanol for 2 min, and finally in water. The sections were stained with hematoxylin for 6 min, rinsed with water, de-colored in 0.1% hydrochloric ethanol for few seconds, and rinsed with water a second time. The sections turned blue in 0.5% ammonia, and were then washed with water three times, followed by staining with eosin for few seconds. The sections were rinsed, briefly placed in 80%, 95%, and 100% ethanol, dehydrated in 100% ethanol for 2 min, and then dried in an incubator at 64°C for 10 min. Next, a drop of neutral gum was placed onto the sample, which was then covered with a cover slip, dried, and sealed. Histomorphological changes in the mouse livers from different groups were examined under an optical microscope

### Pathological examination of mouse tumor tissues

The same method as “Pathological examination of mouse liver tissues”

### Statistical analysis

The results were analyzed by SPSS17.0. A normality test and homogeneity test were performed for all the data. The results were represented by mean ± standard deviation ($\bar{x}\pm s$) and analyzed by a one-way analysis of variance and chi-square test. Analysis of variance was used for comparisons between groups, and *P*<0.05 was considered statistically significant.

Thank Professor Ablez for the guidance of this article.
